# Long‐term immune response to Omicron‐specific mRNA vaccination in mice, hamsters, and nonhuman primates

**DOI:** 10.1002/mco2.460

**Published:** 2023-12-15

**Authors:** Yi Wu, Namei Wu, Xiaoying Jia, Yan Wu, Xinghai Zhang, Yang Liu, Yuxia Hou, Yanqiong Shen, Entao Li, Wei Wang, Yucai Wang, Sandra Chiu

**Affiliations:** ^1^ Department of Laboratory Medicine The First Affiliated Hospital of USTC Division of Life Sciences and Medicine University of Science and Technology of China Hefei Anhui P. R. China; ^2^ Division of Life Sciences and Medicine University of Science and Technology of China Hefei Anhui P. R. China; ^3^ State Key Laboratory of Virology Wuhan Institute of Virology Center for Biosafety Mega‐Science Chinese Academy of Sciences Wuhan P. R. China; ^4^ University of Chinese Academy of Sciences Beijing P. R. China; ^5^ RNAlfa Biotech Hefei Anhui P. R. China; ^6^ Department Key Laboratory of Anhui Province for Emerging and Reemerging Infectious Diseases Hefei Anhui P. R. China; ^7^ Core Unit of National Clinical Research Center for Laboratory Medicine Hefei Anhui P. R. China

**Keywords:** antibody response, immunological memory, long‐term immune response, Omicron subvariants, protective efficacy, SARS‐CoV‐2

## Abstract

Severe acute respiratory syndrome coronavirus 2 (SARS‐CoV‐2) Omicron and its subvariants (such as BQ.1, XBB and the latest variants, including XBB.1.16, EG.5, and BA.2.86), as the dominant variants, currently account for almost all new infections in the world due to their high transmissibility and immune escape ability. Omicron‐specific mRNA vaccines showed great potential to protect against Omicron infections. However, whether the vaccine could provide long‐term protection is unknown. Toward this goal, we evaluated the immunogenicity of a preclinical Omicron (BA.1)‐specific mRNA vaccine (S_Omicron_‐6P) in different animal models. S_Omicron_‐6P induced the highest levels of antibody titers at 1–2 weeks in different animals after the second dose. Even 9 months after the immunization, we observed modest neutralizing activity against Omicron subvariants in macaques. In addition, immunological memory cells can be rapidly reactivated upon stimulation. S_Omicron_‐6P at concentrations higher than 10 μg effectively protected hamsters from BA.1 challenge 253 days after the first immunization, which could be attributed to the reactivation of immune systems. In addition, the toxicity tests conducted in rats revealed a highly favorable biosafety profile for S_Omicron_‐6P, even at high dosages. Our data suggest that the Omicron‐specific mRNA vaccine is highly effective and safe in animal models and provides long‐term immunologic protection against SARS‐CoV‐2 Omicron infections.

## Graphical abstract

Long‐term immune responses to S_Omicron_‐6P were assessed in three animal models. Mice and macaques showed significant decay in antibody titers against BA.1 but remained at high levels around 9 months post‐vaccination. Modest neutralizing antibodies against Omicron subvariants were observed in macaques after 9 months. Immunological memory cells can be rapidly reactivated. S_Omicron_‐6P provides durable protection against Omicron challenge in hamsters.

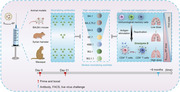

## 1 INTRODUCTION

Severe acute respiratory syndrome coronavirus 2 (SARS‐CoV‐2) Omicron (B.1.1.529), the most recently defined variant of concern (VOC) in the world, was first reported in November 2021 in southern Africa.^1^, ^2^ The Omicron variant is highly contagious and can infect humans more quickly than earlier variants, leading to become the dominant variant in the word. The notable transmissibility among the human population of Omicron variant could be attributed to its highly mutated spike (S) gene.^3^, ^4^ In addition, the abundant mutations in the S protein help them to escape the surveillance of immunity conferred by prior infection or vaccination.^5^, ^6^, ^7^, ^8^, ^9^, ^10^, ^11^, ^12^, ^13^

However, the landscape of SARS‐CoV‐2 pandemic is changing rapidly. The Omicron lineage has continued to evolve and generate new VOCs with additional or different mutations.^12^, ^13^, ^14^ Since the identification of the initial Omicron case in November 2021, an ongoing emergence of new sublineages, ranging from the initial BA.1 to the latest BA.2.86, has triggered successive waves of the pandemic. Among these Omicron variants, BA.1, BA.2, and BA.5 have emerged as predominant strains globally. From January 2023 onward, there has been a swift rise in the prevalence of Omicron BQ.1, along with its sublineages BQ.1.1 (originating from the BA.5 subvariant) and XBB (resulting from a recombination of two BA.2 subvariants) across various countries. This surge has led to the displacement of previously dominant strains such as BA.5.^15^ Most recently, new Omicron subvariants have emerged and spread to numerous countries worldwide, notably XBB.1.16, EG.5, and BA.2.86 (https://www.who.int/activities/tracking‐SARS‐CoV‐2‐variants/). The continued evolution of increased transmissible SARS‐CoV‐2 variants significantly undermines the protective efficacy conferred by historical vaccines targeting the wild‐type (WT) strain of SARS‐CoV‐2 and causes repeat infections around the world that contributes additional risks of death.^16^, ^17^, ^18^

Updated vaccines are highly needed and Omicron‐specific vaccines are likely to have a better protective effect against the Omicron variant. mRNA vaccines have shown great potential in combating SARS‐CoV‐2 infection.^19^ We previously reported a preclinical Omicron‐specific mRNA vaccine that was developed based on the sequence of the S protein of BA.1 and induced six stabilizing proline substitutions (named S_Omicron_‐6P).^20^, ^21^ The vaccine induced up to 27.8‐fold higher serum neutralizing activity against Omicron BA.1 than the WT mRNA vaccine in mice, while the inactivated vaccine and protein subunit vaccine elicited almost no neutralizing activities against BA.1. However, the durability of protection conferred by the Omicron‐specific mRNA vaccine against the homologous virus (BA.1) and its potential for cross‐protection against heterologous viruses (such as BA.5, XBB.1, BQ.1, BQ.1.1, BF.7, and BA.2.75.2) remains uncertain. It was reported that circulating antibodies elicited by clinically approved mRNA vaccines, such as Pfizer BNT162b2 or Moderna mRNA‐1273, declined rapidly within 6 months after the first immunization and exhibited little‐to‐no neutralizing activity against Omicron variants.^22^, ^23^, ^24^, ^25^ A third dose of WT vaccine was needed to recall memory B cells to produce neutralizing antibodies. Infection with an invading virus can also reactivate immunological memory and rebuild antiviral immunity.^23^, ^26^, ^27^, ^28^ However, whether Omicron‐specific mRNA vaccine‐elicited immune memory is efficient enough to combat viral infection is unclear.

The long‐term immune response to vaccines after vaccination is of great importance and determines the vaccination strategy and protective efficacy.^29^, ^30^, ^31^ Moreover, the dynamics of circulating antibodies and long‐term immunity of Omicron‐specific vaccines are poorly understood. Here, we evaluated the long‐term humoral and cellular immune responses and protective efficacy of S_Omicron_‐6P in three different animal models. The circulating antibodies in vaccinated animals declined rapidly and stabilized approximately 4 months after the primary vaccination. Nevertheless, immunization with two doses of S_Omicron_‐6P established both antigen‐specific memory B cells and T cells in the spleen and lymph nodes (LNs), where the lymphocytes could be efficiently reactivated. In addition, the reactivated immune system without a booster vaccination is highly protective against Omicron infections.

## 2 RESULTS

### 2.1 Serum antibody response to S_Omicron_‐6P vaccination in different animal models

**FIGURE 1:**
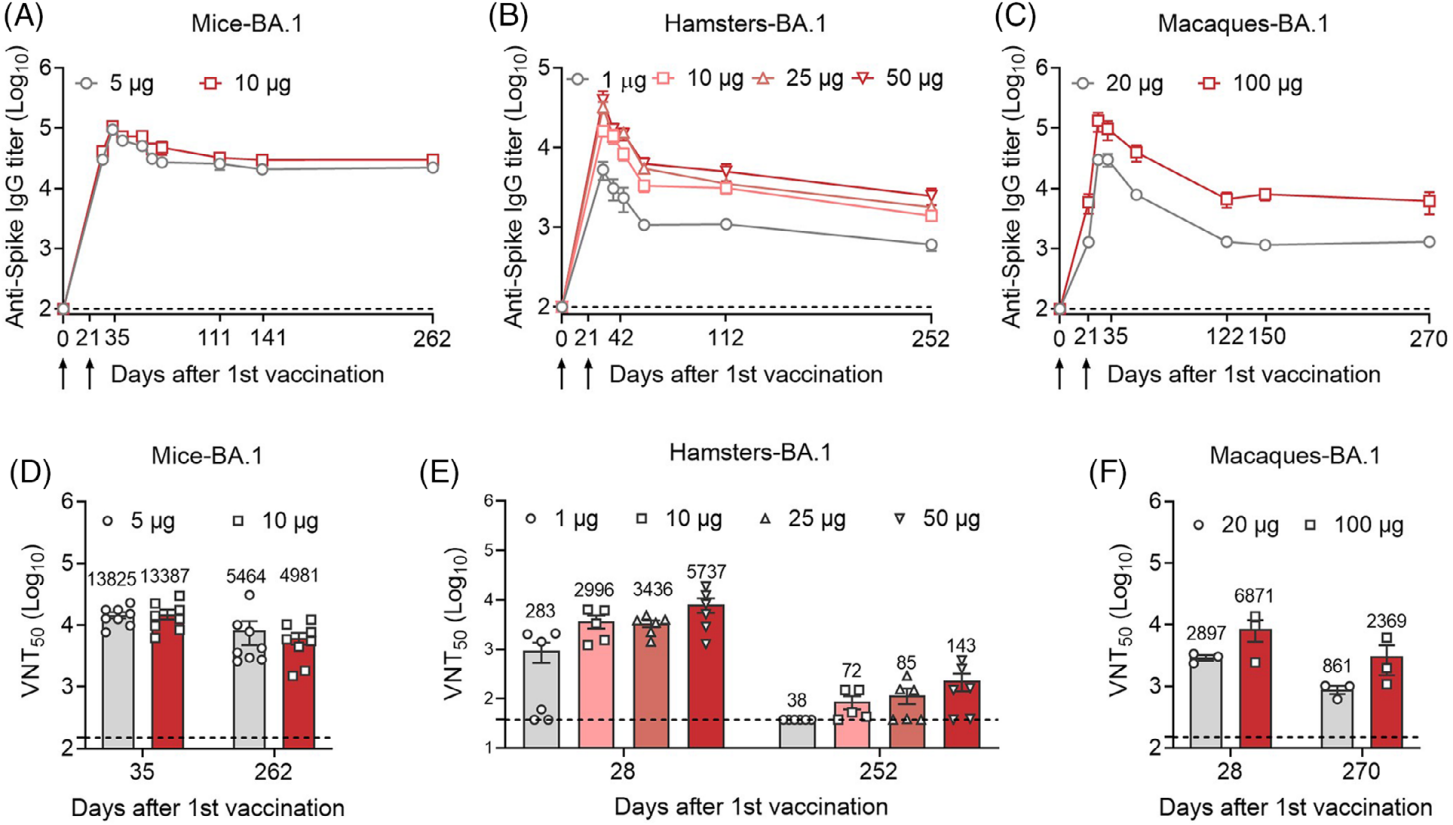
Time course of serum antibody responses against Omicron BA.1 in different vaccinated animal models. (A) Female BALB/c mice were prime‐boost immunized via the intramuscular route with 5 μg (*n* = 8) or 10 μg (*n* = 8) S_Omicron_‐6P on days 0 and 21. Mouse serum was collected on days 0, 28, 35, 42, 56, 70, 111, 141, and 262. The Omicron BA.1‐specific binding antibody titers in serum were determined by  enzyme‐linked immunosorbent assay (ELISA) (lower limit of detection [LLOD] = 100). (B) Female hamsters were prime‐boost immunized via the intramuscular route with 1 μg (*n* = 6), 10 μg (*n* = 5), 25 μg (*n* = 6), or 50 μg (*n* = 6) S_Omicron_‐6P on days 0 and 21. Hamster serum was collected on days 0, 28, 35, 42, 56, 112, and 252. All sera were detected for BA.1‐specific binding antibody titers by ELISA (LLOD = 100). (C) Male macaques were vaccinated intramuscularly with 20 μg (*n* = 3) or 100 μg (*n* = 3) S_Omicron_‐6P on a two‐dose schedule (21‐day interval). The sera of macaques were collected on days 0, 21, 28, 35, 56, 122, 150, and 270. The sera at different time points were detected for BA.1‐specific binding antibody titers by ELISA (LLOD = 100). (D) The 50% virus‐neutralization titers (VNT_50_) against SARS‐CoV‐2 Omicron BA.1 in mice on days 35 and 262 were determined by a plaque reduction neutralization test (PRNT) (LLOD = 150). (E) VNT_50_ against BA.1 in hamsters on days 28 and 252 (LLOD = 150 for day 28 and 37.5 for day 252). (F) VNT_50_ against BA.1 in macaques on days 28 and 270 (LLOD = 150). The heights of the bars indicate the geometric means for each group, and the values expressed as geometric means are presented above the bars. All data are shown as the mean ± SEM.

BALB/c mice were prime‐boost injected with 5 or 10 μg S_Omicron_‐6P on a two‐dose schedule (21‐day interval), and serum samples were harvested from day 0 (before vaccination) to day 262 (Figure 1A). BA.1‐specific binding antibody kinetics showed that S_Omicron_‐6P induced the highest level of antibody response on day 35 (2 weeks from the second immunization). The binding antibody titers stabilized ∼111 days after primary vaccination and remained at approximately 23.5% (the 5 μg group) and 26.4% (the 10 μg group) of the peak level (Figures 1A and S1A). In Syrian hamsters, animals were immunized with 1, 10, 25, or 50 μg S_Omicron_‐6P on days 0 and 21 (Figure 1B). Hamster serum was harvested and determined on days 0, 28, 35, 42, 56, 112, and 252. We observed the highest level of BA.1‐specific binding antibody titers on day 28 (1 week from the second immunization). Unlike in mice, the binding antibody titers in hamster serum decreased with time instead of stabilizing after a certain time (Figures 1B and S1B). Furthermore, we evaluated the binding antibody kinetics in nonhuman primates (NHPs). Two groups of macaques were injected intramuscularly with 20 and 100 μg of S_Omicron_‐6P on days 0 and 21 (Figure 1C). Consistent with the results in mice, the highest binding antibody titers were observed on day 28 (1 week from the second immunization), which stabilized ∼122 days after the first vaccination and remained at approximately 7.9% (the 20 μg group) and 17.9% (the 100 μg group) of the peak level (Figures 1C and S1C).

After characterizing the binding antibody responses in different vaccinated animal models over time, we wondered whether the remaining antibodies in the serum could neutralize homologous SARS‐CoV‐2 Omicron infections. We thus analyzed the corresponding neutralizing activities against authentic Omicron BA.1 by a plaque reduction neutralization test (PRNT). As shown in Figures 1D and S2, in the mouse model, circulating neutralizing antibody titers against BA.1 virus remained high at ∼9 months after primary vaccination, although there was an approximately 60.5%–62.8% reduction compared to the peak levels. Sharply, in the hamster models, the neutralizing antibodies against BA.1 dropped to very low levels at ∼8 months after primary vaccination, an approximately 86.7%–97.9% reduction compared to the peak levels (Figures 1E and S3). Similar to the results in the mouse models, the data in macaques revealed a reduction in neutralizing antibody titers against BA.1 ranging from 65.5% to 70.3% compared to the peak levels (Figures 1F and S4).

**FIGURE 2:**
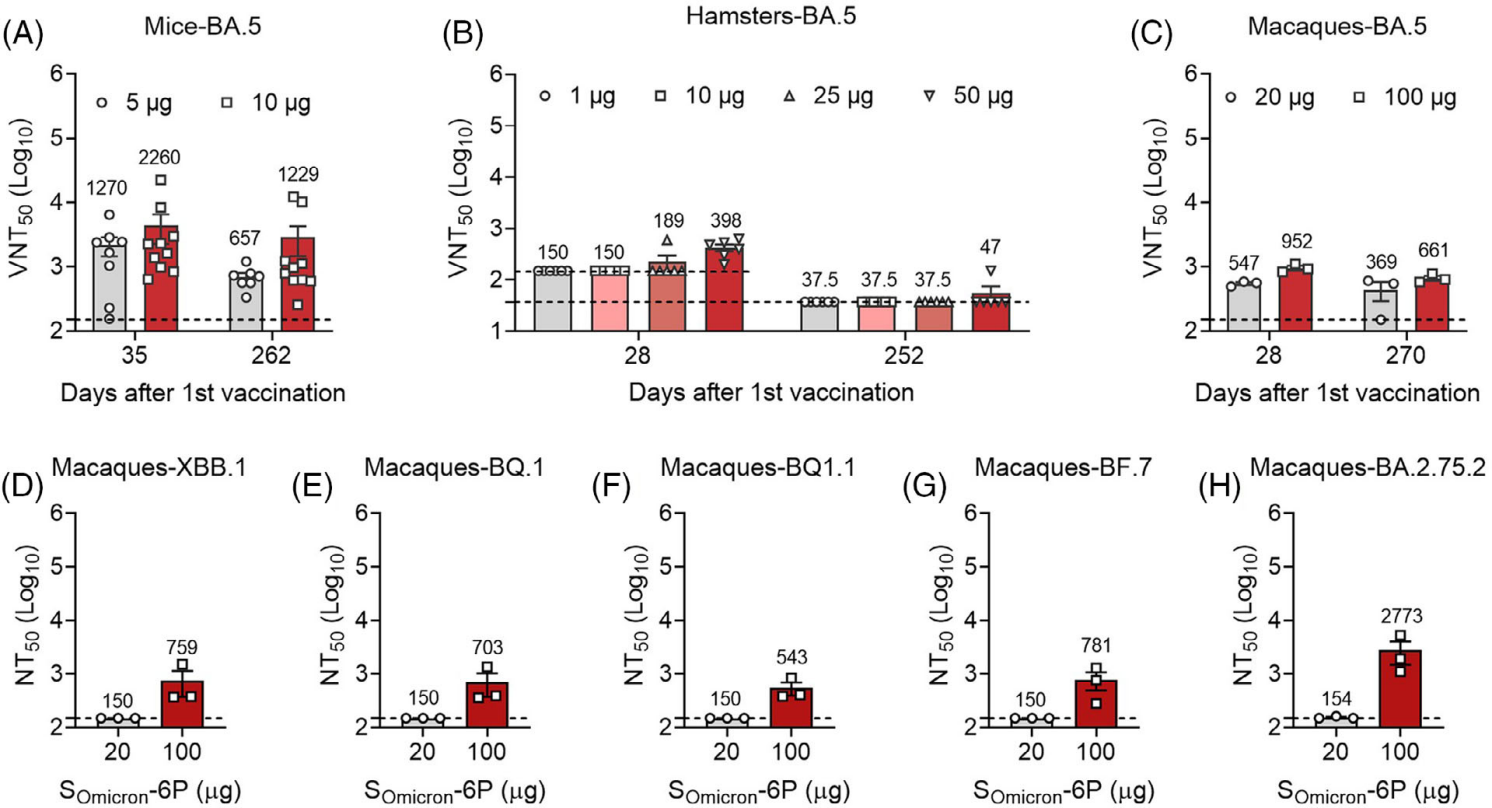
Long‐term antibody activities elicited by S_Omicron_‐6P vaccination against heterologous Omicron subvariants. (A) The 50% virus‐neutralization titers (VNT_50_) against BA.5 in mice (*n* = 8) on days 35 and 262 (lower limit of detection [LLOD] = 150). (B) VNT_50_ against BA.5 in hamsters (*n* = 6 for the 1, 25, and 50 μg groups, and *n* = 5 for the 10 μg group) on days 28 and 252 (LLOD = 150 for day 28 and 37.5 for day 252). (C) VNT_50_ against BA.5 in macaques (*n* = 3) on days 28 and 270 (LLOD = 150). (D–H) Neutralization titers (NT50) against XBB.1 (D), BQ.1 (E), BQ.1.1 (F), BF.7 (G), and BA.2.75.2 (H) in macaques on day 270 were measured by a pseudovirus neutralization assay (LLOD = 150). The heights of the bars indicate the geometric means for each group, and the values expressed as geometric means are presented above the bars. All data are shown as the mean ± SEM.

As the Omicron lineage continued to evolve, a series of subvariants with additional or different mutations were identified. BA.5, as the sublineage of Omicron, is rapidly becoming predominant in various countries and is characterized by distinct humoral immune escape patterns.^13^ Compared to Omicron BA.1, the neutralizing activities against BA.5 decreased more significantly in the three animal models (Figures 2A–C and S5–S7). Specifically, S_Omicron_‐6P elicited modest geometric mean titers (GMTs) against BA.5 in mice (1270 for 5 μg and 2260 for 10 μg on day 35; 657 for 5 μg and 1229 for 10 μg on day 262) and macaques (547 for 20 μg and 952 for 100 μg on day 28; 369 for 20 μg and 661 for 100 μg on day 270) but not hamsters. Additionally, numerous novel subvariants emerged as dominant strain in 2023. Consequently, we studied the neutralization activities against other new Omicron subvariants in macaques on day 270 by using a pseudovirus neutralization assay (Figures 2D–H). Undetectable levels of neutralizing antibodies against XBB.1, BQ.1, BQ.1.1, BF.7, and BA.2.75.2 were observed in macaques immunized with two doses of 20 μg S_Omicron_‐6P, while the neutralization titers were still detectable (ranging from 543 to 2773) in the group immunized with 100 μg S_Omicron_‐6P 9 months after primary vaccination. Given that Omicron subvariants demonstrated a significantly greater capacity for humoral immune escape compared to BA.1 and few vaccines exhibited excellent protection against these subvariants, we speculate that the Omicron‐specific vaccine may confer durable cross‐protection against Omicron subvariants.^11^, ^12^, ^13^, ^32^

### 2.2 Both B cells and T cells were reactivated specifically by stimulation with the antigen peptide pool

**FIGURE 3:**
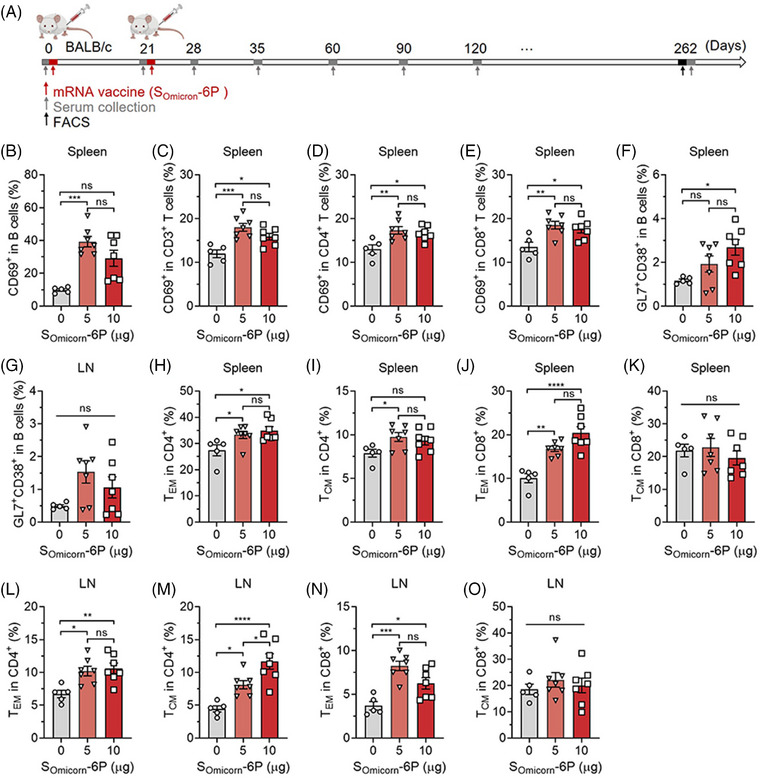
S_Omicron_‐6P elicited long‐lasting immune memory against Omicron in mice. (A) Schematic showing the procedure of the mouse experimentation. Mice were injected intramuscularly with two doses of 0 μg (*n* = 5), 5 μg (*n* = 7), or 10 μg (*n* = 7) S_Omicron_‐6P on days 0 and 21. The serum was collected at the indicated time points and determined for a specific antibody response. On day 262, all the mice were sacrificed, and the spleen and draining lymph nodes (LNs) (popliteal and inguinal LNs) were collected and homogenized for singlets. The obtained lymphocytes were stimulated with a spike (S) protein peptide pool for 12 h. After stimulation, the cells were analyzed for cell phenotyping by flow cytometry. (B–E) Flow cytometric analysis of activated B cells (CD69^+^CD3^−^B220^+^) (B), total T cells (CD69^+^CD3^+^) (C), CD4^+^ (CD69^+^CD3^+^CD4^+^) (D), and CD8^+^ T cells (CD69^+^CD3^+^CD8^+^) (E) in the spleen. (F and G) The percentages of plastic CD38^+^GL7^+^ B‐cell precursors in the spleen (F) and LNs (G). (H–K) The percentages of effector memory T cells (T_EM_, CD44^+^CD62L^−^) and central memory T cells (T_CM_, CD44^+^CD62L^+^) in CD4^+^ and CD8^+^ T cells in the spleen. (L–O) The percentages of T_EM_ and T_CM_ in CD4^+^ and CD8^+^ T cells in the LNs. Data are displayed as the mean ± SEM. Statistics were conducted using unpaired one‐way analysis of variance (ANOVA) with multiple comparison tests. ^*^ *p* < 0.05, ^**^ *p* < 0.01, ^***^ *p* < 0.001, ^****^ *p* < 0.0001; ns, not significant.

The immune response gradually declines to normal levels after the second dose of vaccination.^33^, ^34^, ^35^, ^36^ As shown in Figure 1, antibody titers reached peak levels 1 or 2 weeks after the second vaccination and declined rapidly thereafter. To determine whether the immune system can be rapidly reactivated upon stimulation with the antigens, we harvested splenocytes and draining LN cells from different vaccinated mice and stimulated these cells with an S peptide pool for 12 h (Figure 3A). After stimulation, the cells were stained with fluorescent antibodies and analyzed by flow cytometry. Compared to the 0 μg group, activated (CD69^+^) B cells, CD3^+^ T cells, and CD4^+^ and CD8^+^ T cells were significantly elevated in the spleen, which was not observed in the LNs (Figures 3B–E and S8–S9). These results indicated that splenic lymphocytes in animals after a long time from full vaccination can be rapidly recalled to an activated state when encountering the same antigens. Deeply, the results also reflected that draining LNs may play an important role in the early stage after mRNA vaccine immunization, while the long‐term rapid immune responses could be more dependent on the spleen.^37^, ^38^ Interestingly, the results also showed that a lower activation of B and T lymphocytes in mice vaccinated with the highest dose. By analyzing the changes in proportions and mean fluorescence intensity, we observed that both CD4^+^ and CD8^+^ T cells are subject to the negative feedback regulation from the high‐dose vaccine, with CD4^+^ T cells appearing to be more significantly affected. However, the underlying mechanisms remain unclear and require further investigation. In addition, we identified an increased percentage of CD38^+^GL7^+^ B‐cell precursors in both the spleen and LNs of vaccinated mice (Figure 3F,G). CD38^+^GL7^+^ B cells, as multipotent precursors, can differentiate into memory B cells and later germinal center (GC) B cells.^39^, ^40^, ^41^ We also observed extensive expansion of effector memory (T_EM_, CD44^+^CD62L^−^) and center memory (T_CM_, CD44^+^CD62L^+^) CD4^+^ and CD8^+^ T cells in both the spleen and LNs (Figures 3H–O and S10), which mediate protective cellular immune memory.

### 2.3 Antigens recall a Th1‐biased immune response in vaccinated animals

**FIGURE 4:**
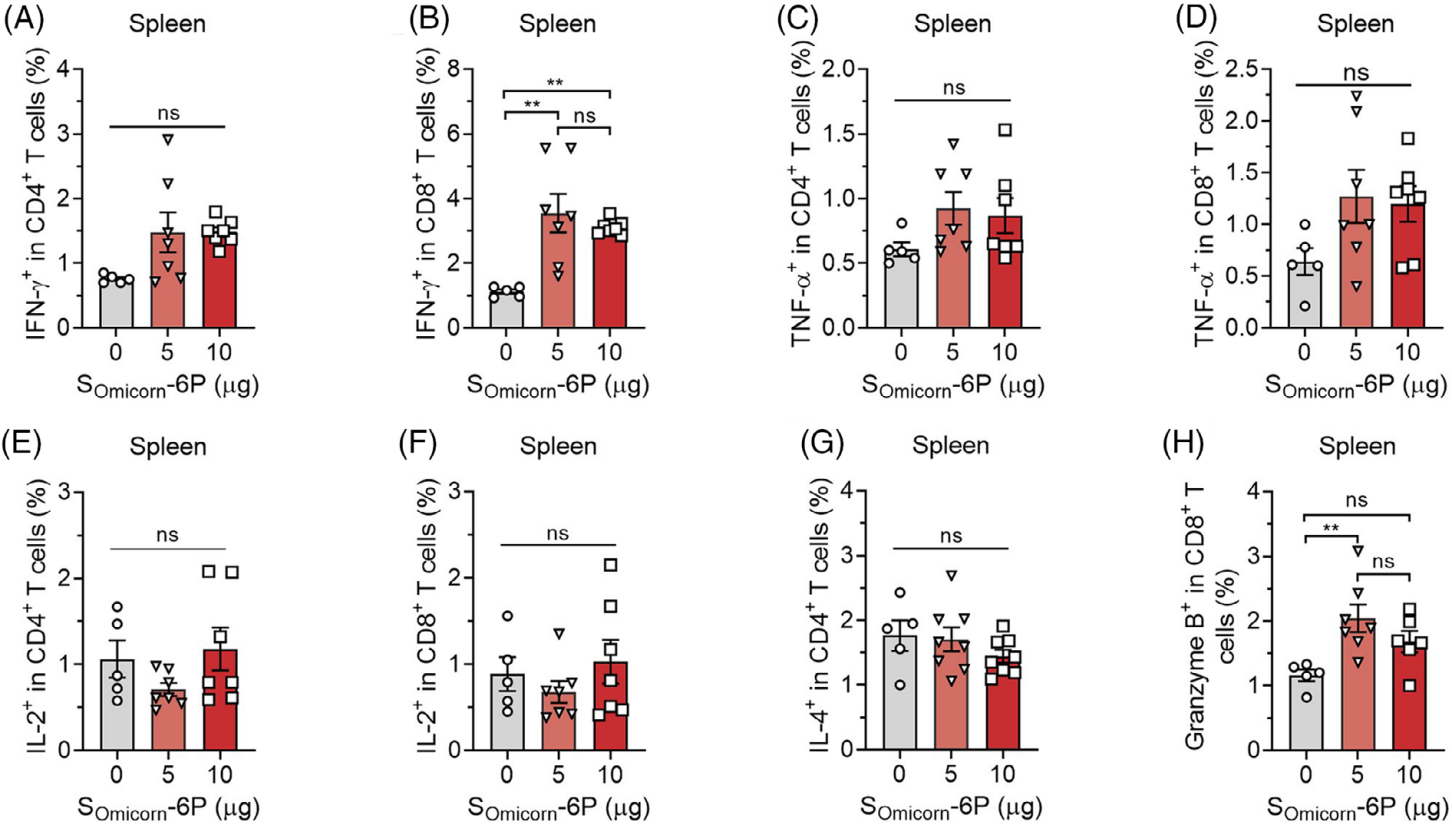
Th1‐biased immune response to the antigen peptide pool in vaccinated mice. (A–F) Peptide pool‐stimulated splenic T cells of different immunized groups were stained for type 1 interferon‐gamma (IFN‐γ) (A and B), tumor necrosis factor‐alpha (TNF‐α) (C and D), and interleukin‐2 (IL‐2) (E and F) intracellular cytokine expression in CD4^+^ T cells and CD8^+^ T cells. (G) The expression of type 2 IL‐4 intracellular cytokines in CD4^+^ T cells. (H) The frequency of the cytolytic marker granzyme B expressed in CD8^+^ T cells. Data are shown as the mean ± SEM. *n* = 5 for the 0 μg group, *n* = 7 for the 5 and 10 μg groups. Statistics were calculated using unpaired one‐way analysis of variance (ANOVA) with multiple comparison tests. ^*^ *p* < 0.05, ^**^ *p* < 0.01; ns, not significant.

To further determine the functional immune response in mice on day 262, we analyzed the expression of type 1/type 2 intracellular cytokines and cytolytic markers after peptide pool stimulation. Compared to the 0 μg group, immunization with 5 or 10 μg S_Omicron_‐6P revealed a higher fraction of CD4^+^ and CD8^+^ T cells that secreted interferon‐gamma (IFN‐γ) but not tumor necrosis factor‐alpha (TNF‐α) or interleukin‐2 (IL‐2) (Figures 4A–F and S11). Three groups of mice vaccinated with or without S_Omicron_‐6P produced non‐distinctive levels of IL‐4 (Figure 4G). Moreover, a higher proportion of CD8^+^ T cells produced the cytolytic marker granzyme B (Figure 4H). Intracellular cytokine data confirmed that S_Omicron_‐6P elicited a higher frequency of CD4^+^ T cells that produced IFN‐γ, but no fluctuation in the frequency of CD4^+^ T cells that produced IL‐4 indicated a Th1‐biased immune response.^42^

### 2.4 S_Omicron_‐6P elicited long‐term protection in hamsters

**FIGURE 5:**
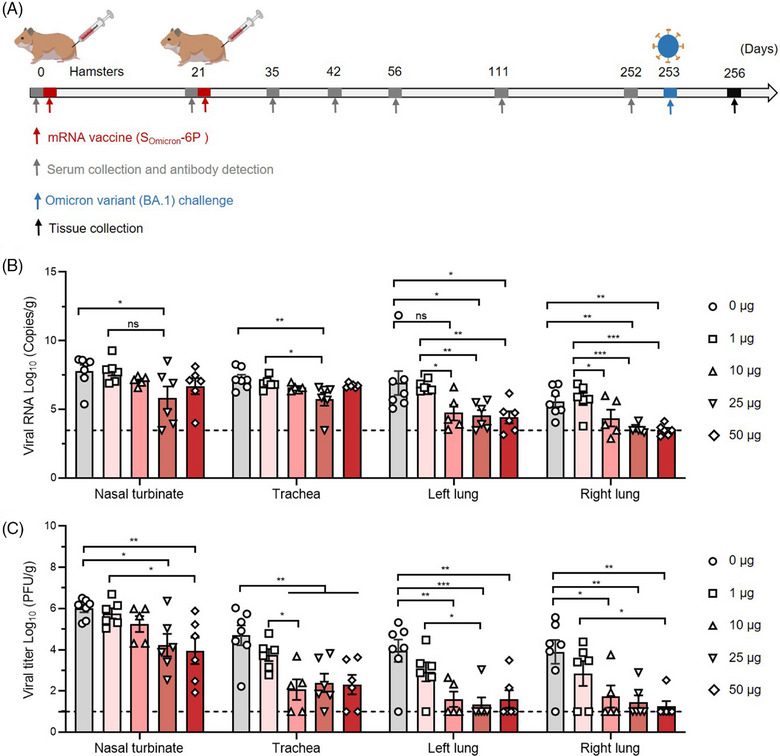
S_Omicron_‐6P generated long‐term protective immunity against Omicron infections in hamsters. (A) Study design. Syrian hamsters were injected intramuscularly with two doses of 0 μg (*n* = 7), 1 μg (*n* = 6), 10 μg (*n* = 5), 25 μg (*n* = 6), or 50 μg (*n* = 6) S_Omicron_‐6P on days 0 and 21. Hamster serum was collected at the indicated time points, and the specific antibody response was determined. At 253 days after the first vaccination, hamsters of all groups were challenged intranasally with Omicron BA.1. After three days, animals were sacrificed for tissue collection and further analysis. (B) Viral RNA loads in the nasal turbinate, trachea, and both lungs were determined by quantitative reverse transcription‐polymerase chain reaction (qRT‒PCR). (C) Viral loads expressed in tissues (including all above mentioned tissues). Data are shown as the mean ± SEM. Statistics were calculated using unpaired one‐way analysis of variance (ANOVA) with multiple comparison tests. ^*^ *p* < 0.05, ^**^ *p* < 0.01, ^***^ *p* < 0.001; ns, not significant.

As demonstrated above, elicited immune responses decayed rapidly to a steady state in different animal models after two doses of vaccination. Approximately 8 months after primary vaccination, circulating antibodies exhibited significantly lower levels of neutralizing activities against Omicron. However, S_Omicron_‐6P established long‐lasting immune memory in vivo. The immune system of vaccinated animals can be efficiently reactivated by the peptide pool ex vivo. We next investigated the long‐term protective efficacy in hamster models. Different vaccinated hamsters were challenged intranasally with 1 × 10^4^ plaque‐forming units (PFU) SARS‐CoV‐2 Omicron on day 253 (Figure 5A). On day 3 after infection, the tissues were harvested and further analyzed. Compared to the 0 and 1 μg groups, 10–50 μg S_Omicron_‐6P greatly reduced the viral RNA loads in both lungs, and some of the animals were even detected with no viral RNA (Figure 5B). In close agreement with viral RNA loads, high viral loads were measured by plaque assay in the nasal turbinate, trachea, left lungs, and right lungs of the 0 and 1 μg S_Omicron_‐6P vaccinated hamsters (Figure 5C). In comparison, markedly lower levels of live virus were detected in these tissues of the 10, 25, and 50 μg vaccinated groups. In both lungs, 40% (2/5) of animals in the 10 μg group, 66.7% (4/6) of animals in the 25 μg group, and 66.7% (4/6) of animals in the 50 μg group had no live virus. However, the observed low levels of neutralizing antibody titers at day 252 were insufficient to account for the robust long‐term protection observed in hamsters. We speculate that the long‐term protection against Omicron infections in hamsters could be partly attributed to immune memory elicited by S_Omicron_‐6P.

### 2.5 The levels of antibody titers correlate with protection against Omicron infection

**FIGURE 6:**
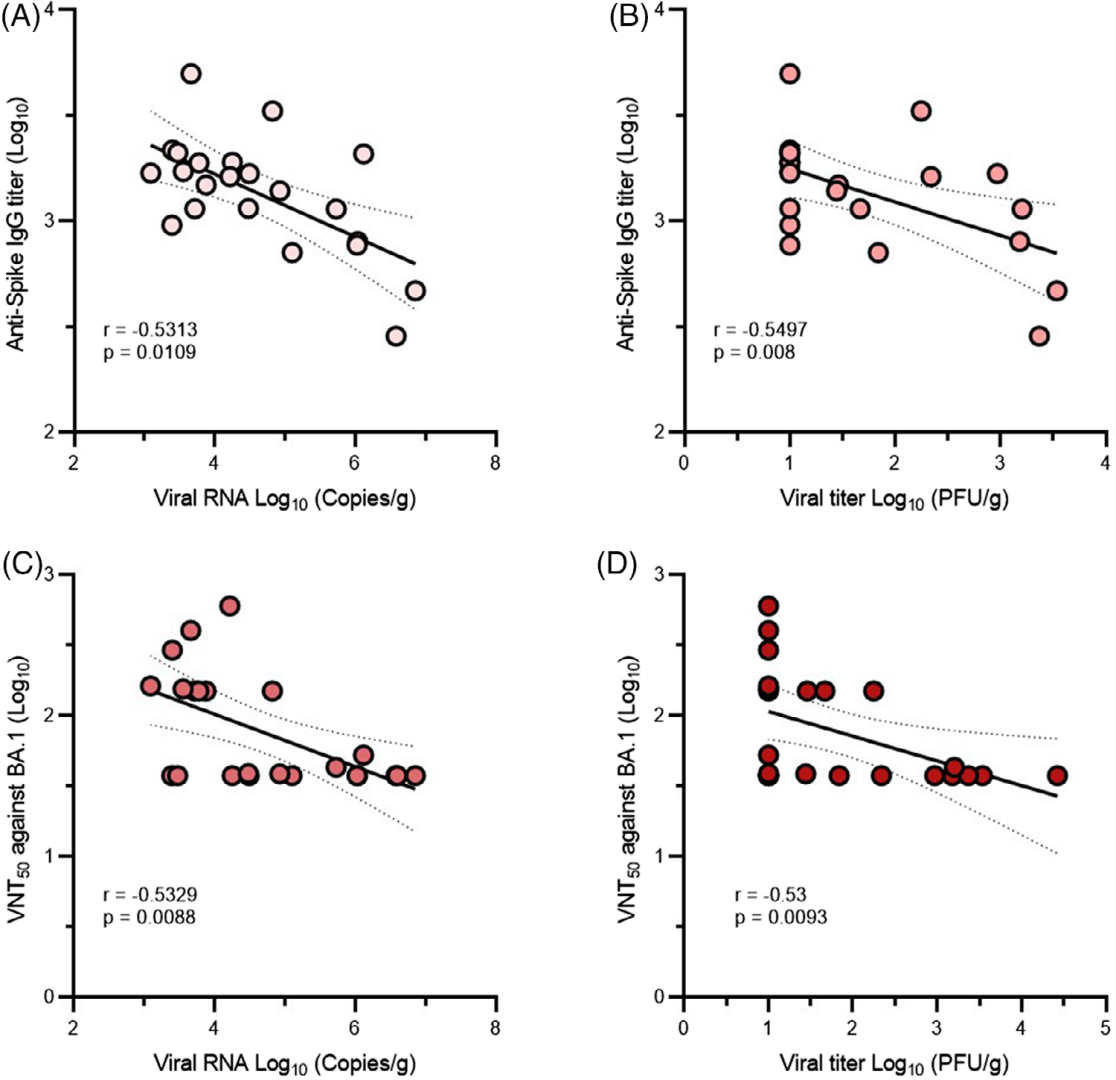
Correlations of antibody responses and protection against Omicron BA.1 in S_Omicron_‐6P‐vaccinated hamsters. For all correlations, immunoglobulin G (IgG) titers and neutralizing antibody titers against Omicron BA.1 in hamsters extracted from Figure 1, and viral loads in lungs of hamsters were obtained from Figure 5 (*n* = 23). (A and B) Spearman correlations between IgG titers against Omicron BA.1 (on day 252) and viral RNA loads (A) as well as live viral titers (B) in hamster lungs. (C and D) Spearman correlations between neutralizing antibody titers against Omicron BA.1 (on day 252) and viral RNA loads (C) as well as live viral titers (D) in hamster lungs. Spearman's correlation coefficients (*r*) and *p*‐values (*p*) are shown in the graphs.

We then studied whether the remained antibody levels correlate with protective efficacy of S_Omicron_‐6P in hamsters. The correlations between viral loads and antibody responses in hamsters were analyzed. As shown in Figure 6A,B, inverse correlations between immunoglobulin G (IgG) titers and viral RNA loads (*r* = −0.5313, *p* = 0.0109) as well as IgG titers and live viral loads (*r* = −0.5497, *p* = 0.008) were observed. Similarly, 50% virus‐neutralization titers (VNT_50_) titers were also inversely correlated with lung viral RNA load (*r* = −0.5329, *p* = 0.0088) and live viral titer (*r* = −0.53, *p* = 0.0093) (Figure 6C,D). These results suggest that the antibody response induced by S_Omicron_‐6P can serve as immune correlates of protection against Omicron infection. The inverse correlations between antibody responses and viral loads indicate that S_Omicron_‐6P would confer cross‐protection against Omicron subvariants, as evidenced by detectable cross‐neutralization observed in macaques at 9 months.

### 2.6 S_Omicron_‐6P exhibits excellent biocompatibility and safety profiles in vivo

**FIGURE 7:**
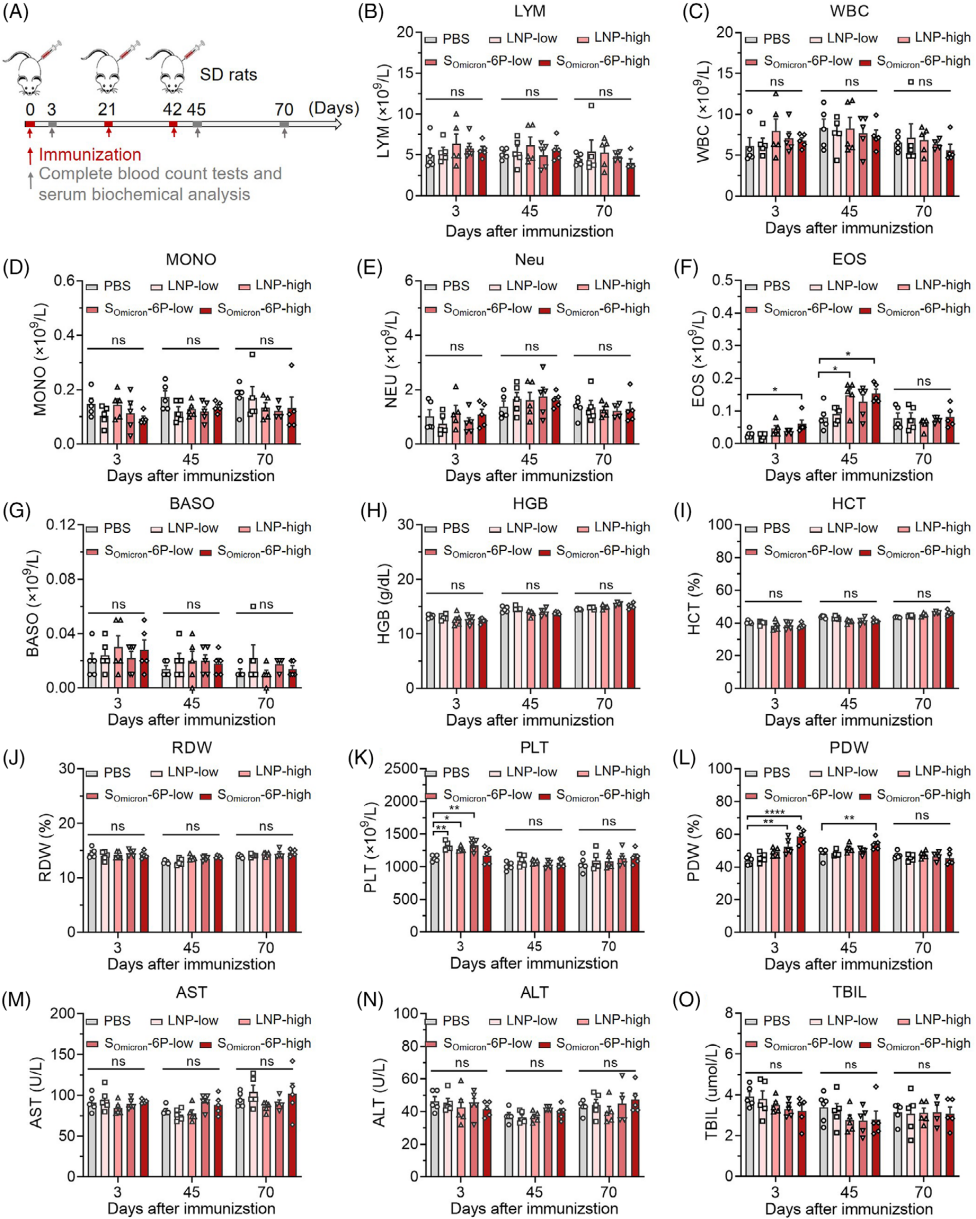
Biosafety evaluation of S_Omicron_‐6P in Sprague–Dawley (SD) rats. (A) Male rats (*n* = 5) were intramuscularly immunized with phosphate buffered saline (PBS), lipid nanoparticle (LNP) (low and high dosages), and S_Omicron_‐6P (low and high dosages) on days 0, 21, and 42. Low and high dosages of LNP were abbreviated as LNP‐low and LNP‐high, respectively. These formulations had an equivalent total LNP content compared to the corresponding vaccine groups but lacked mRNA. Low and high dosages of S_Omicron_‐6P contained 30 and 60 μg mRNA that abbreviated as S_Omicron_‐6P‐low and S_Omicron_‐6P‐high. On days 3, 45, and 70, both blood and serum were collected for complete blood count (CBC) tests and serum biochemical analysis. (B–L) CBC tests. (M–O) Serum biochemical analysis. Data are displayed as the mean ± SEM. Statistics were conducted using unpaired one‐way analysis of variance (ANOVA) with multiple comparison tests. ^*^ *p* < 0.05, ^**^ *p* < 0.01, ^****^ *p* < 0.0001; ns, not significant. ALT, alanine aminotransferase; AST, aspartate aminotransferase; BASO, basophils; EOS, eosinophils; HCT, hematocrit; HGB, hemoglobin; LYM, lymphocytes; MONO, monocytes; Neu, neutrophils; PDW, platelet distribution width; PLT, platelets; RDW, red cell distribution width; TBIL, total bilirubin; WBC, white blood cells.

The tentative optimal dosage of S_Omicron_‐6P for adults has been established at 30 μg/0.3 mL. To further evaluate the toxicity of the Omicron‐specific mRNA vaccine, we immunized rats three times with either an equivalent or a twofold human dosage of the vaccine and analyzed its biosafety at different time points (Figure 7A). The data from complete blood count tests demonstrated that high dosages of lipid nanoparticle (LNP) or S_Omicron_‐6P can lead a certain inflammatory response, manifested by elevated eosinophils. The local inflammatory response at the administration site can disrupt the animals' normal physiological habits, leading to mild anemia, manifested by elevated platelets and platelet distribution width (Figure 7B–L). However, these symptoms completely disappeared 28 days after the final immunization. In addition, no abnormal results were observed in the serum biochemistry analysis (Figure 7M–O). The above data were obtained from male rats and a similar phenomenon was observed in female rats as well (data are not shown). These results suggested that S_Omicron_‐6P, even at high dosages, did not exhibit significant toxic side effects in animals. Therefore, the vaccine demonstrates great biocompatibility and biosafety.

## 3 DISCUSSION

SARS‐CoV‐2 Omicron and its subvariants currently predominate worldwide. Although Omicron variant infection causes less severe symptoms in adults, it is still dangerous for elderly people and those with weak immunity. In addition, the virus can cause all kinds of long‐term health symptoms and sequelae in infected individuals, which are poorly understood. Immunization with an effective vaccine has been proven to significantly decrease the incidence of sequelae induced by SARS‐CoV‐2 infection. However, Omicron escapes the neutralizing antibodies conferred by prior vaccines. We previously reported an Omicron‐specific mRNA vaccine that exhibited high immunogenicity and protective efficacy in the short term. The protective effects of vaccines can wane over time due to declining immunologic memory. However, data regarding the long‐term immune responses, protective efficacy, and biosafety profiles of the Omicron‐specific mRNA vaccine are still lacking.

We reasoned that validating the dynamics of circulating antibodies and long‐term immunologic memory in different animal models (including NHPs) would provide beneficial information for Omicron‐specific mRNA vaccination in the clinic. We present several key pieces of data demonstrating that S_Omicron_‐6P elicited durable immunity against Omicron and its subvariants. S_Omicron_‐6P induced peak levels of antibody titers in different animals at 1–2 weeks after the second dose. The circulating antibody titers declined rapidly but stabilized between 4 and 9 months post‐vaccination in BALB/c mouse and macaque models. However, the antibody titers in hamsters continued to decline after reaching peak levels. Nonetheless, in three different models, a higher dose of S_Omicron_‐6P corresponded to higher antibody titers at any time after vaccination, which indicates that the intensity but not the duration of humoral immune responses depends on the dose of S_Omicron_‐6P. Different from mice and macaques (that exhibited similar and durable antibody response), hamsters even immunized with very high dose of S_Omicron_‐6P exhibited weak antibody response, indicating that hamster may be not a good animal model for long‐term humoral immunity evaluation for Omicron‐specific vaccines. Moreover, we observed significantly decreased but acceptable levels of neutralizing activities against Omicron subvariants (including BA.5, XBB.1, BQ.1, BQ.1.1, BF.7, and BA.2.75.2), indicating the cross‐protection potential of S_Omicron_‐6P.

An effective vaccine builds up long‐lasting immune memory, which can be recalled to a reactivated state. We observed reactivated B cells and T cells in the spleen but not the LNs of S_Omicron_‐6P‐vaccinated mice at approximately 9 months after primary vaccination. The results indicate that the spleen rather than LNs may play a more important role in long‐term antiviral immunity, which highlights the risks in patients with splenectomy. Significantly higher percentages of memory T cells and B‐cell precursors were observed in both the spleen and LNs of vaccinated mice. Memory lymphocytes rapidly differentiate into functional immune cells that help combat viral infection. Further immunology analysis identified that antigens still recalled a Th1‐biased immune response long after full vaccination. In vivo live BA.1 challenge results confirmed the long‐term protective efficacy conferred by S_Omicron_‐6P, which may be contributed by the vaccine‐established memory immune system. In addition, the toxicity tests performed in rats demonstrated that S_Omicron_‐6P has excellent biocompatibility and safety profiles in vivo

In summary, the Omicron‐specific mRNA vaccine S_Omicron_‐6P exhibited significant potential in combating Omicron and its subvariants across various models. The immunological memory built up by two doses of S_Omicron_‐6P can be efficiently recalled to an activated state by antigen stimulation. However, there are several limitations to the current work. The circulating antibody titers wane rapidly to negligible levels after reaching peak levels. SARS‐CoV‐2 infection long after vaccination may be related to the lack of neutralizing antibodies. A third dose of S_Omicron_‐6P would highly recall immunological memory and boost circulating antibody levels, but the correlates of protection efficacy against Omicron and its subvariants are still being defined. High amount of vaccine could contribute to the levels and durability of immunity, which deserves more exploratory work. In addition, it is unclear whether memory B cells or T cells play more important roles in protecting against viral infections. Nonetheless, this study highlights the potential for long‐term immunological memory induced by a preclinical Omicron‐specific mRNA vaccine and the latent long‐term immunologic protection against Omicron infections, which helps enable a better understanding of vaccine immunobiology in humans.

## 4 MATERIALS AND METHODS

### 4.1 Study design

The objective of this study was to evaluate the long‐term immune response and protective efficacy of an Omicron‐specific mRNA based on the “hexapro” backbone in three different animal models, including mice, hamsters, and macaques. Starting January 8, 2022, three models of animals were established to be vaccinated with different doses of S_Omicron_‐6P, and the corresponding humoral and cellular immune responses and protective efficacy were examined after approximately 9 months. Omicron S‐specific antibody responses were monitored in the three animal models for the whole time. The recall immune responses in mice were analyzed by flow cytometry upon stimulation with the S peptide pool. Durable protection against SARS‐CoV‐2 Omicron was investigated in hamsters by challenge with the authentic virus. Viral loads in different organs were determined by plaque assay and quantitative reverse transcription‐polymerase chain reaction (qRT‒PCR).

### 4.2 Virus and cell lines

Omicron BA.1 (CCPM‐B‐V‐049‐2112‐18) and BA.5 (NPRC2.062200006) were kindly obtained from the National Pathogen Resource Center. Experiments that involved authentic viruses were conducted in a biosafety level‐3 facility. The Vero E6 cells used in this research were obtained from American Type Culture Collection (Cat# CRL‐1586).

### 4.3 mRNA vaccine

Omicron‐specific mRNA vaccine design and production were previously described.^15^ Briefly, the vaccine was developed based on the sequence of the S protein of Omicron BA.1 with six stabilizing proline substitutions (F817P, A892P, A899P, A942P, K986P, and V987P). mRNA was synthesized via in vitro transcription reactions. Then, the purified mRNA was encapsulated in LNPs using microfluidics. The LNP contains an ionizable amino lipid, heptadecan‐9‐yl 8‐{(2‐hydroxyethyl) [6‐oxo‐6‐(undecyloxy) hexyl] amino} octanoate (SM‐102), a PEGylated lipid, 1,2‐dimyristoylrac‐glycero‐3‐methoxypolyethylene glycol‐2000 (DMG‐PEG2000) and two structural lipids (1,2‐distearoyl‐sn‐glycero‐3‐phosphocholine and cholesterol). After analytical characterization, including mRNA encapsulation, size, pH, and endotoxin, the product was deemed for in vivo study.

### 4.4 Mice

BALB/c mice (8–12 weeks) were immunized intramuscularly with 5 or 10 μg S_Omicron_‐6P in the hind leg on days 0 and 21, and mice that received phosphate buffered saline (PBS) served as a negative control. Blood was collected on days 0, 28, 35, 42, 56, 63, 70, 111, 141, and 262 after the primary immunization. Mice were sacrificed on day 262, and their spleens and LNs were collected for reactivation immune response evaluation by flow cytometry.

### 4.5 Hamsters

Syrian hamsters (6–7 weeks) were immunized intramuscularly with 1, 10, 25, or 50 μg S_Omicron_‐6P on days 0 and 21, and hamsters that received PBS served as a negative control. Blood was collected on days 0, 28, 35, 42, 56, 112, and 252 after the first immunization. Animals were challenged intranasally with 1 ×10^4^ PFU of Omicron BA.1 on day 253. Lungs, nasal turbinates, and tracheas were collected on day 256 for viral load analysis as described below.

### 4.6 Macaques

Two groups of macaques were immunized with two doses of 20 or 100 μg S_Omicron_‐6P. Blood was collected on days 0, 21, 28, 35, 56, 122, 150, and 270 after the first immunization. Binding and neutralizing antibody titers were assessed by enzyme‐linked immunosorbent assay (ELISA) and PRNT.

### 4.7 IgG titers (ELISA)

Spike‐specific binding antibodies in serum were tested using ELISA as previously described.^43^ Ninety‐six‐well ELISA plates (Thermo Fisher) were precoated with 0.1 μg of Omicron BA.1 spike protein (Sino Biological) in 1× PBS buffer per well overnight at 4°C. After incubation, the plates were slapped dry and blocked with 5% skim milk powder in PBST for 2 h at room temperature. The plates were washed with PBST four times after each step. All heat‐inactivated sera were diluted in blocker buffer. Following blocking, serial threefold dilutions of sera (starting at 1:100) were added to plates and incubated for 1 h at room temperature. Next, a 1‐h incubation with a secondary antibody conjugated with horseradish peroxidase (HRP) at room temperature was carried out. Plates were slapped dry and washed, and 100 μL Tetramethylbenzidine (TMB) buffer (Beyotime) was added to the plates and incubated for 8 min at room temperature. After incubation, the TMB–HRP reaction was halted by adding 50 μL of stop solution (Beyotime). The optical density at 450 nm was measured by a microplate reader (SpectraMax iD5, Molecular Devices).

### 4.8 VNT_50_ assay (PRNT)

SARS‐CoV‐2 Omicron BA.1 and BA.5 were used to determine the neutralizing activities as previously described with modifications.^15^ Briefly, serial fourfold dilutions of heat‐inactivated sera (starting at 1:37.5 or 1:150) were incubated with the authentic virus for 1 h at 37°C. Subsequently, the virus–antibody mixtures were added in duplicate to Vero E6 cells preseeded in 24‐well plates and incubated for another 1 h at 37°C. Following incubation, the virus–antibody mixtures were replaced with medium that contained 0.9% carboxymethyl‐cellulose, and the plates were then incubated for 3 days at 5% CO_2_ and 37°C. The plates were further fixed with 8% paraformaldehyde (PFA) and stained with 1% crystal violet to visualize the infected cell foci. The VNT_50_ titers were calculated by GraphPad Prism 8.0.1 software.

### 4.9 Flow cytometry

For T‐cell and B‐cell subtyping, extracellular antigens in lymphocytes derived from LNs and spleen were stained with fluorescent dye‐conjugated antibodies. After blocking with anti‐CD16/CD32 blockade (BD Biosciences), the cells were then stained with two panels of monoclonal antibodies for 30 min at 4°C. Panel 1: Brilliant Violet 421 anti‐mouse CD3 (BD Pharmingen, Cat# 562600), PerCP/Cyanine5.5 anti‐mouse B220 (Biolegend, Cat# 562600), APC/Fire 750 anti‐mouse CD19 (Biolegend, Cat# 115557), PE/Cyanine7 anti‐mouse CD69 (BD Pharmingen, Cat# 552879), FITC anti‐mouse GL7 (BD Pharmingen, Cat# 553666), and APC anti‐mouse CD38 (Biolegend, Cat# 102712). Panel 2: Brilliant Violet 421 anti‐mouse CD3 (BD Pharmingen, Cat# 562600), APC anti‐mouse CD4 (Biolegend, Cat# 100412), APC/Cyanine7 anti‐mouse CD8a (Biolegend, Cat# 100714), PE/Cyanine7 anti‐mouse CD69 (BD Pharmingen, Cat# 552879), Brilliant Violet 510 anti‐mouse CD62L (Biolegend, Cat# 104441), and PE/Cyanine5 anti‐mouse/human CD44 (BD Pharmingen, Cat# 553135). Cells were acquired by using the CytoFLEX LX flow cytometer, and the data were analyzed with the associated CytoFLEX LX software.

For intracellular cytokine staining, 5 × 10^6^ live splenocytes were restimulated ex vivo with an overlapping peptide mix (a total of 313 peptides) that spans full‐length S protein (Sino Biological) for 12 h at 5% CO_2_ and 37°C. After restimulation, the cells were then stained with the following two panels of monoclonal antibodies for 30 min at 4°C. Panel 1: Brilliant Violet 421 anti‐mouse CD3 (BD Pharmingen, Cat# 562600), PE anti‐mouse CD8a (BD Pharmingen, Cat# 553032), and FITC anti‐mouse CD4 (Biolegend, Cat# 100406). Panel 2: Brilliant Violet 421 anti‐mouse CD3 (BD Pharmingen, Cat# 562600), APC/Cyanine7 anti‐mouse CD8a (Biolegend, Cat# 100714), and PE/Cyanine7 anti‐mouse CD4 (Biolegend, Cat# 100422). After washing, the cells were fixed, permeabilized, and stained intracellularly with the corresponding antibody panel for another 30 min. Panel 1: PE/Cyanine7 anti‐mouse IFN‐γ (BD Pharmingen, Cat# 557649) and APC anti‐mouse TNF‐α (Biolegend, Cat# 506308). Panel 2: APC anti‐mouse IL‐2 (Biolegend, Cat# 503810), PE anti‐mouse IL‐4 (Biolegend, Cat# 504104), and FITC anti‐human/mouse granzyme B (Biolegend, Cat# 396404). Cells were acquired by using the CytoFLEX LX flow cytometer, and the data were analyzed with the associated CytoFLEX LX software.

### 4.10 Viral RNA measurement (qRT‒PCR)

To determine the viral loads in different organs of infected hamsters, viral RNA was measured by qRT‒PCR as previously reported.^44^ Briefly, viral RNA was extracted from nasal turbinate, trachea, and lung tissue homogenates, purified with a QIAamp Viral RNA Mini Kit (Qiagen) and stored at −80°C for further use. Viral RNA quantification was determined by using a qRT‒PCR SYBR Green Kit (Vazyme Biotech) with two primers. The detection sensitivity was determined to be 120 copies per gram of the tissue samples. The following primers were used in the qRT‒PCR assay: ORF1a/b—forward: (5′‐CCCTGTGGGTTTTACACTTAA‐3′) and ORF1a/b—reverse (5′‐ACGATTGTGCATCAGCTGA‐3′).

### 4.11 Analysis of viral load by plaque assay

Viral titers were tested by plaque assay. At 3 days post‐infection, nasal turbinate, trachea, and lung tissues were collected, homogenized and clarified. The samples were serially diluted tenfold in DMEM. Subsequently, the serial dilutions were added to adherent Vero E6 cells preseeded in 24‐well plates and incubated for 1 h at 5% CO_2_ and 37°C. Following incubation, the supernatants were replaced with medium that contained 0.9% carboxymethyl‐cellulose, and the plates were then incubated for 3 days at 5% CO_2_ and 37°C. The plates were further fixed with 8% PFA and stained with 1% crystal violet to visualize the infected cell foci. The infected cell foci were recorded (10–100 plaques) and calculated for virus titers.

### 4.12 Statistical analysis

Flow cytometric data were analyzed using Beckman CytoFlex software. All statistical analyses and relative graphs were performed using GraphPad Prism version 8.0.1. Data are displayed as the mean ± SEM. Analysis of variance or *t*‐test was used to determine statistical significance among different groups (^*^ *p* < 0.05; ^**^ *p* < 0.01; ^***^ *p* < 0.001; ^****^ *p* < 0.0001).

## AUTHOR CONTRIBUTIONS

The project was conceived by S.C., Y.‐C.W., Yi W., and N.‐M.W. The ELISAs and T/B‐cell analysis were carried out by Yi W., N.‐M.W., and Y.‐Q.S. Authentic SARS‐CoV‐2 experiments were performed by X.‐Y.J., Yan W., X.‐H.Z., Y.L., Y.‐X.H., and E.‐T.L. Static analysis was performed by Yi W., N.‐M.W., X.‐Y.J., and Yan W. The original draft was written by Yi W., N.‐M.W., Y.‐C.W., and S.C. Review and editing were performed by W.W., Y.‐C.W., and S.C. All authors have read and approved the final manuscript.

## CONFLICT OF INTEREST STATEMENT

Yucai Wang is the inventor on pending patent applications related to the Omicron mRNA vaccine. The remaining authors declare they have no conflicts of interest.

## ETHICS STATEMENT

Mouse and macaque experiments were performed under protocols approved by the Institutional Animal Care and Use Committee of the USTC (approval no. USTCACUC25010122049). Syrian hamster studies were approved by the Animal Ethics Committee of the Wuhan Institute of Biological Products (WIBP) (WIBP‐AII382020001).

## Supporting information

Supporting information

## Data Availability

Upon request, the corresponding author will provide the relevant data.
