# Supplementary material for: Long‐term immune response to Omicron‐specific mRNA vaccination in mice, hamsters, and nonhuman primates
Source: MedComm (2020). 2023 Dec 15;4(6):e460. doi: 10.1002/mco2.460 (PMC10724501; doi:10.1002/mco2.460)
Supplement: Supplementary file 1 — Supporting information [file MCO2-4-e460-s001.docx]

Supplementary Materials for

**Long-term immune response to Omicron-specific mRNA vaccination in mice, hamsters, and nonhuman primates**

Yi Wu^1,2#^, Namei Wu^1#^, Xiaoying Jia^3#^, Yan Wu^3#^, Xinghai Zhang^3^, Yang Liu^3^, Yuxia Hou^3,4^, Yanqiong Shen^5^, Entao Li^1,2,7^, Wei Wang^3,4^*, Yucai Wang^2,5^*, Sandra Chiu^1,2,6,7^*

^1^Department of Laboratory Medicine, The First Affiliated Hospital of USTC, Division of Life Sciences and Medicine, University of Science and Technology of China, Hefei, Anhui, 230031, P. R. China.

^2^Division of Life Sciences and Medicine, University of Science and Technology of China, Hefei, Anhui 230027, P. R. China.

^3^State Key Laboratory of Virology, Wuhan Institute of Virology, Center for Biosafety Mega-Science, Chinese Academy of Sciences, Wuhan, 430062, P. R. China.

^4^University of Chinese Academy of Sciences, Beijing, 100049, P. R. China.

^5^RNAlfa Biotech, Hefei, Anhui 230088, P. R. China.

^6^Core Unit of National Clinical Research Center for Laboratory Medicine, Hefei, Anhui, 230031, P. R. China.

^7^Key Laboratory of Anhui Province for Emerging and Reemerging Infectious Diseases, Hefei, 230027, P. R. China

^#^These authors contributed equally: Yi Wu, Namei Wu, Xiaoying Jia, Yan Wu.

***Correspondence:**

Wei Wang, State Key Laboratory of Virology, Wuhan Institute of Virology, Center for Biosafety Mega-Science, Chinese Academy of Sciences, Wuhan, 430062, P. R. China.

Wei Wang: [wangwei@wh.iov.cn](mailto:wangwei@wh.iov.cn)

Yucai Wang, Division of Life Sciences and Medicine, University of Science and Technology of China, Hefei, Anhui 230027, P. R. China.

Email: [yucaiwang@ustc.edu.cn](mailto:yucaiwang@ustc.edu.cn);

Sandra Chiu, Department of Laboratory Medicine, The First Affiliated Hospital of USTC, Division of Life Sciences and Medicine, University of Science and Technology of China, Hefei, Anhui, 230031, P. R. China.

Email: [qiux@ustc.edu.cn](mailto:qiux@ustc.edu.cn)


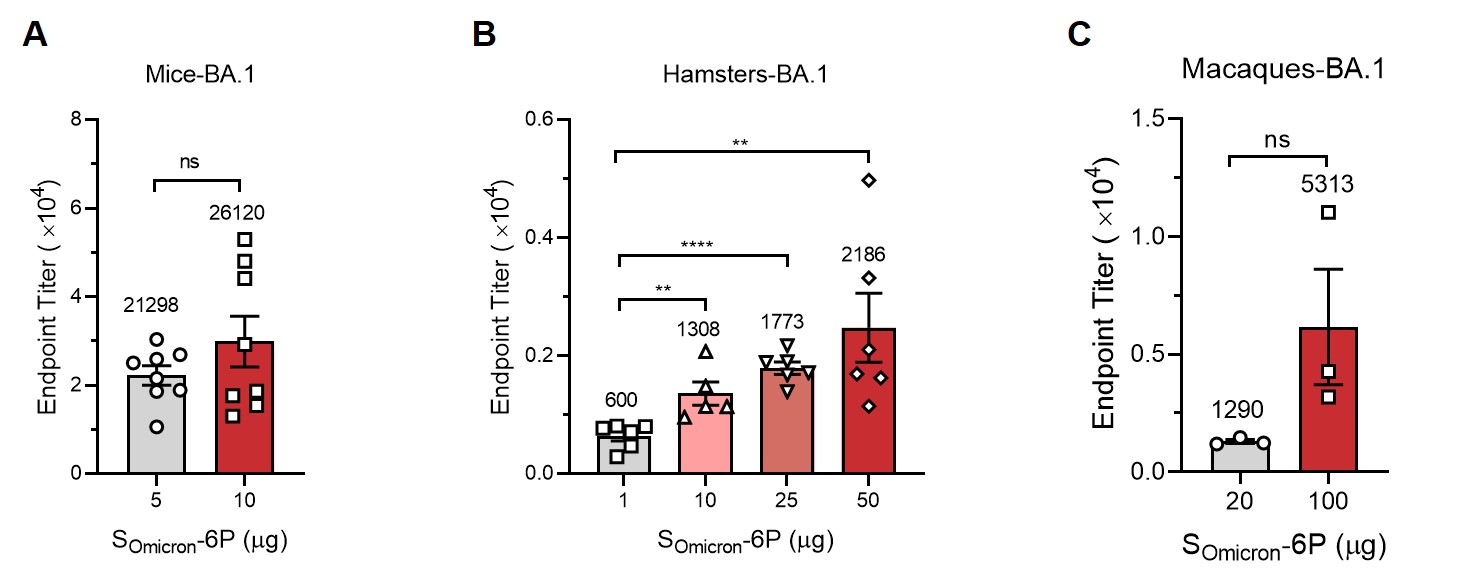


**FIGURE S1. Serum binding antibody response in different animal models on the endpoint day.** (A-C) The Omicron (BA.1) spike-specific IgG titers in mouse serum (A) collected on day 262 (n = 8), hamster serum (B) collected on day 252 (n = 6 for the 1 μg, 25 μg, and 50 μg groups, and n = 5 for the 10 μg group), and macaque serum (C) collected on day 270 (n = 3).


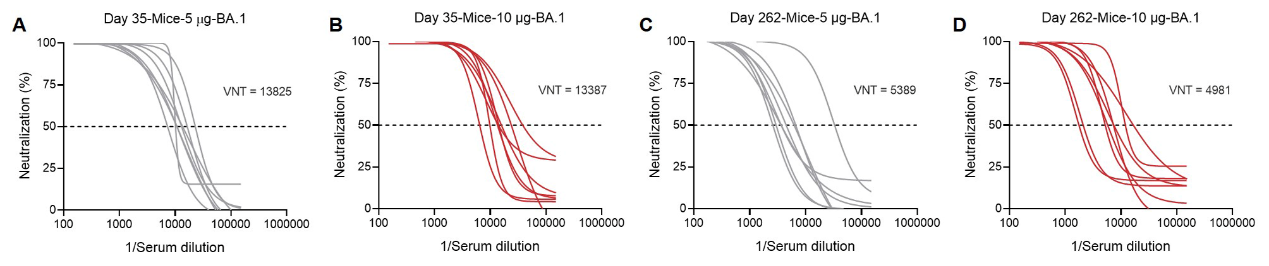


**FIGURE S2. The 50% virus-neutralization titers (VNT_50_) against BA.1 in mice on day 35 and day 262.** (A-D) Serum neutralizing activities against BA.1 were detected by a plaque reduction neutralization test (PRNT) on day 35 (A-B) and day 262 (C-D). Neutralization curves corresponding to individual mice that received 5 μg (n =8) or 10 μg (n = 8) S_Omicron_-6P.


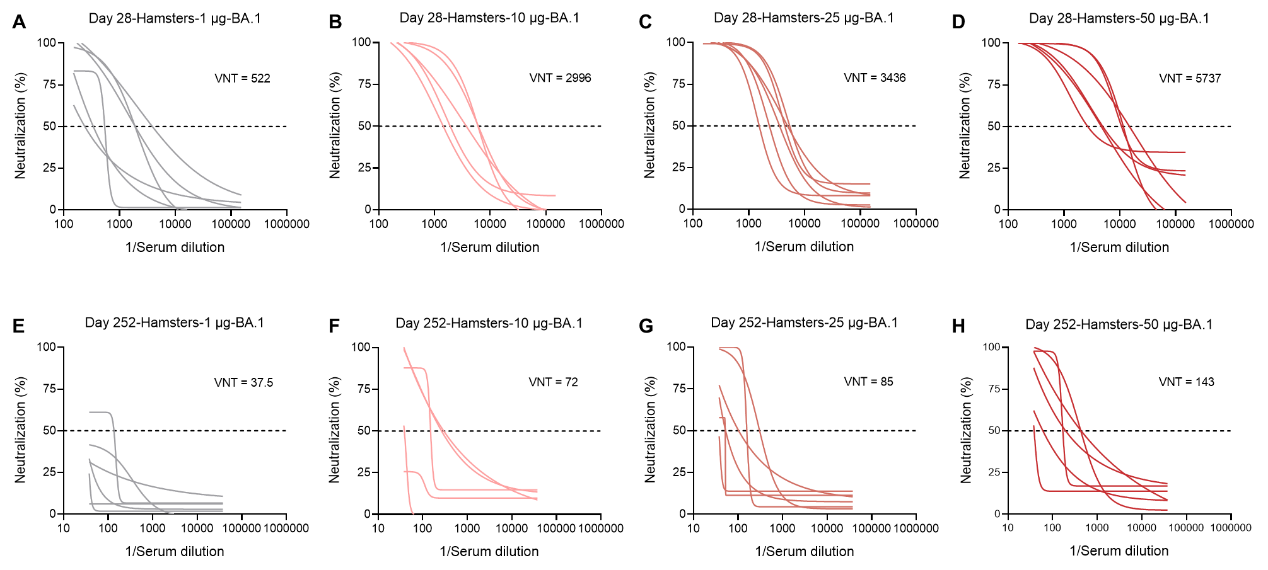


**FIGURE S3. VNT_50_ against BA.1 in hamsters on day 35 and day 252.** (A-H) Serum neutralizing activities against BA.1 were detected by PRNT on day 28 (A-D) and day 252 (E-H). Neutralization curves corresponding to individual hamsters that received 1 μg (n = 6), 10 μg (n = 5), 25 μg (n = 6), or 50 μg (n = 6) S_Omicron_-6P.


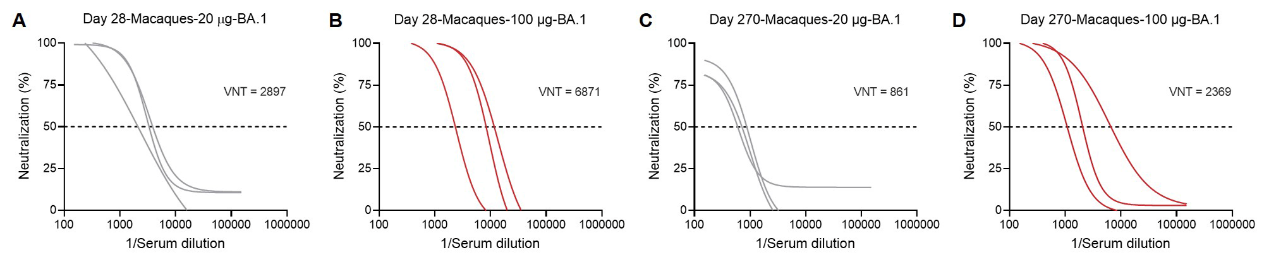


**Figure S4. VNT_50_ against BA.1 in macaques on day 28 and day 270.** (A-D) Serum neutralizing activities against BA.1 were detected by PRNT on day 28 (A-B) and day 270 (C-D). Neutralization curves corresponding to individual macaques that received 20 μg (n = 3) or 100 μg (n = 3) S_Omicron_-6P.


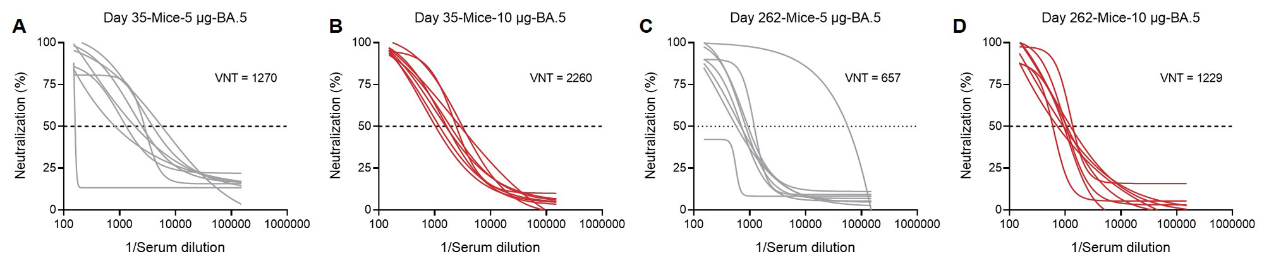


**Figure S5. VNT_50_ against BA.5 in mice on day 35 and day 262.** (A-D) Serum neutralizing activities against BA.5 were detected by PRNT on day 35 (A-B) and day 262 (C-D). Neutralization curves corresponding to individual mice that received 5 μg (n = 8) or 10 μg (n = 8) S_Omicron_-6P.


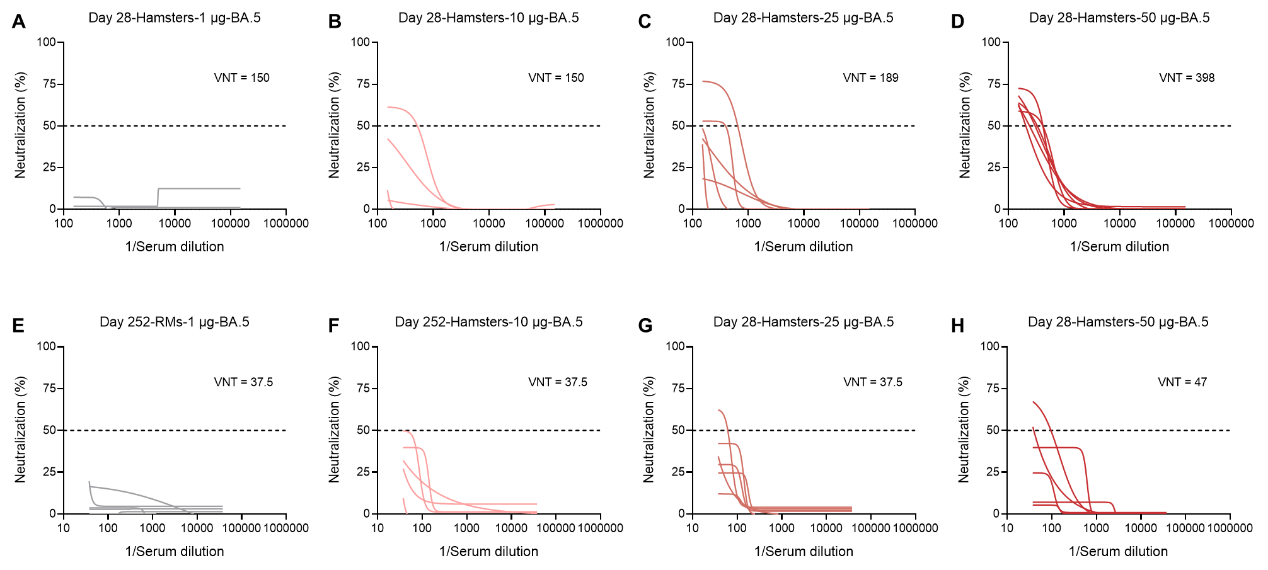


**Figure S6. VNT_50_ against BA.5 in hamsters on day 35 and day 252.** (A-D) Serum neutralizing activities against BA.5 were detected by PRNT on day 28 (A-D) and day 252 (E-H). Neutralization curves corresponding to individual hamsters that received 1 μg (n = 6), 10 μg (n = 5), 25 μg (n = 6), or 50 μg (n = 6) S_Omicron_-6P.


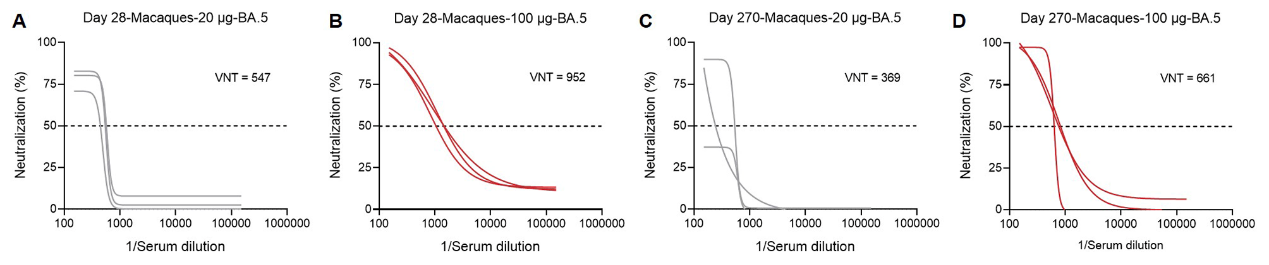


**Figure S7. VNT_50_ against BA.5 in macaques on day 28 and day 270.** (A-D) Serum neutralizing activities against BA.5 were detected by PRNT on day 28 (A-B) and day 270 (C-D). Neutralization curves corresponding to individual macaques that received 20 μg (n = 3) or 100 μg (n = 3) S_Omicron_-6P.


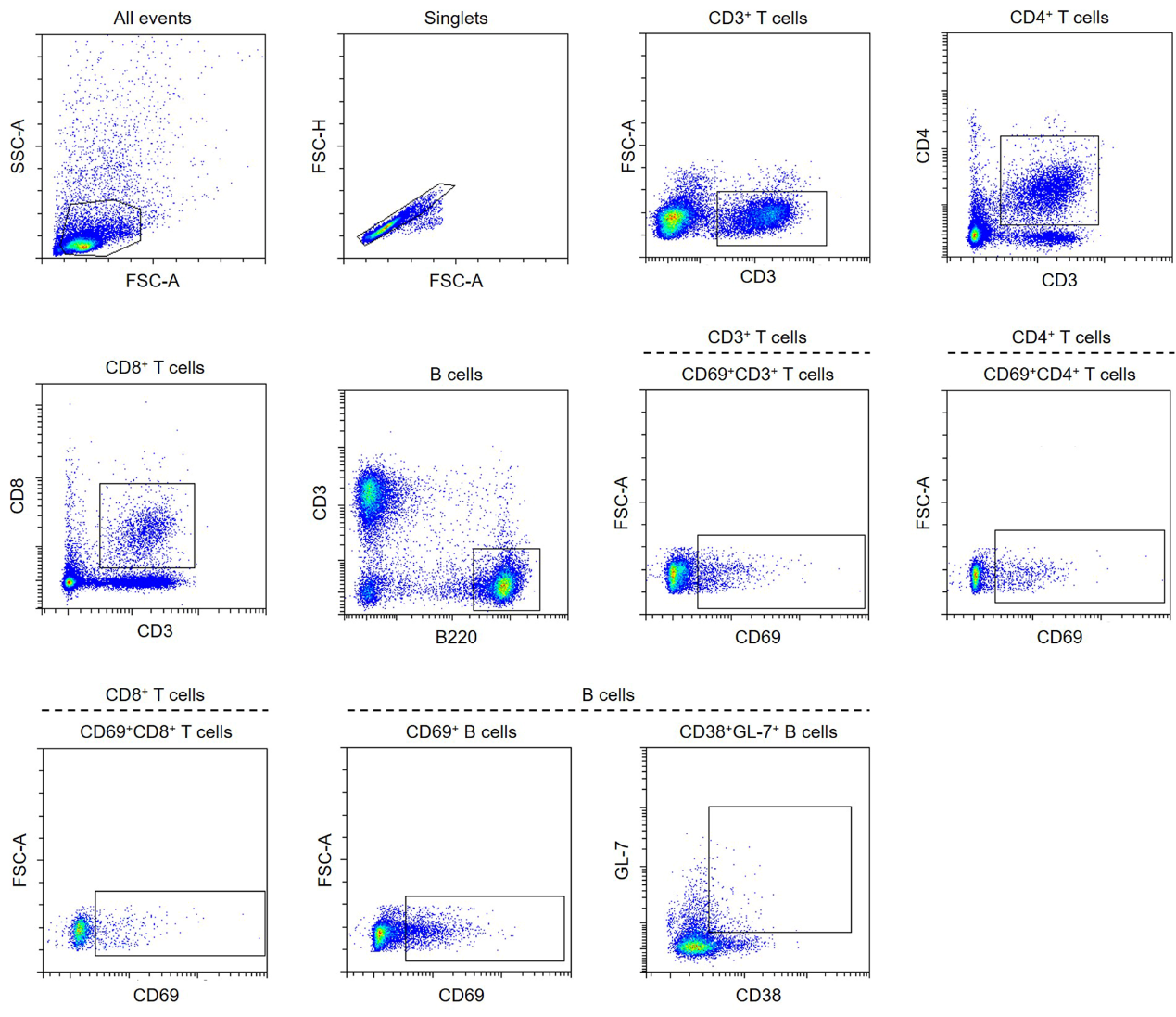


**FIGURE S8. The gating strategy for flow cytometry analysis of data is shown in Figure 2A-G and Figure S9.** Flow cytometry gating strategy for the identification of activated B cells, T cells, and plastic CD38^+^GL7^+^ B-cell precursors.


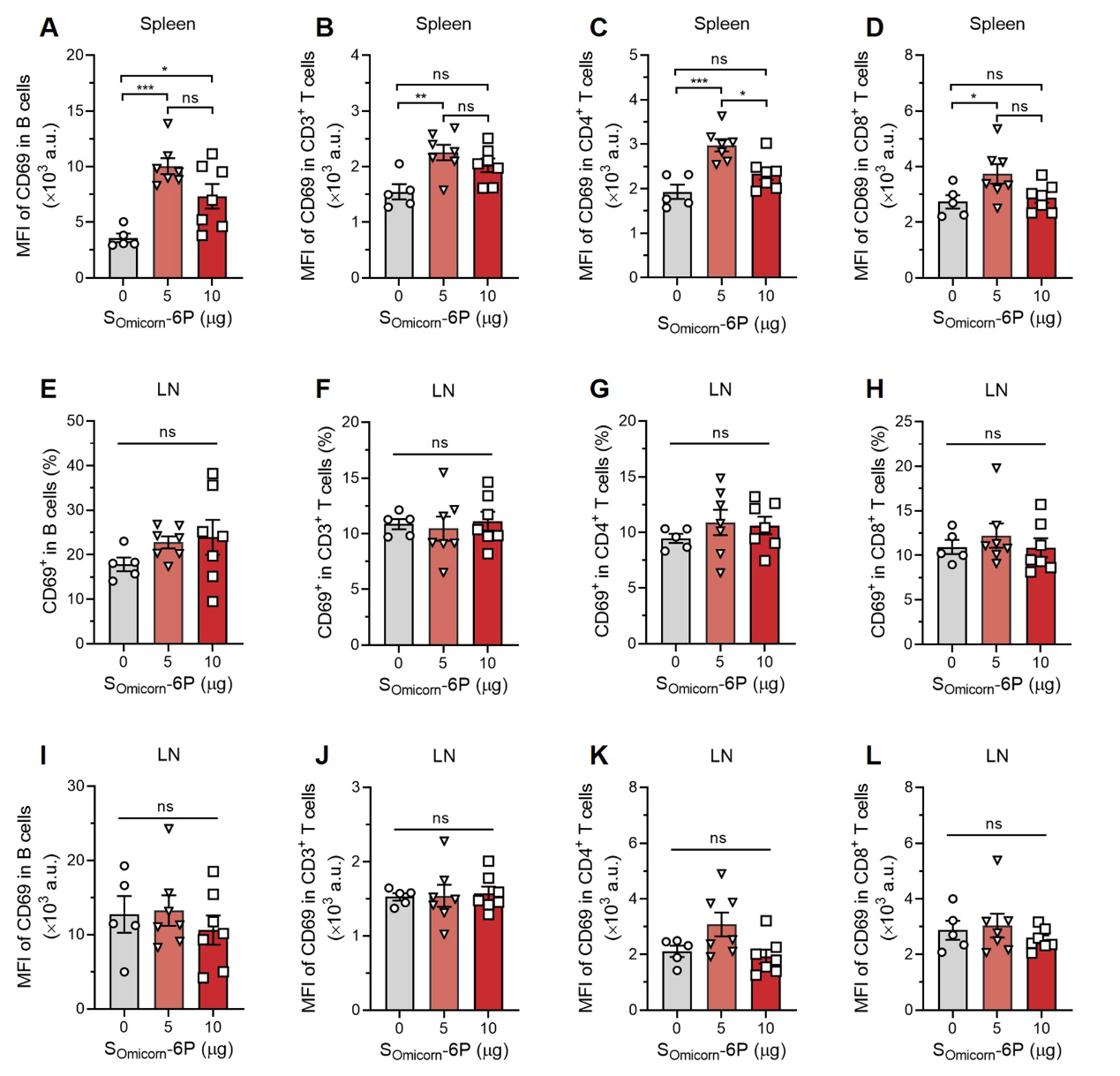


**FIGURE S9. Analysis of reactivated immune cells in spleen and LNs.** (A-D) Mean fluorescence intensity (MFI) of CD69 in B cells (A), total T cells (B), CD4^+^ (C), CD8^+^ (D) T cells in the spleen. (E-H) Flow cytometric analysis of activated B cells (CD69^+^CD3^-^B220^+^) (E), total T cells (CD69^+^CD3^+^) (F), CD4^+^ T cells (CD69^+^CD3^+^CD4^+^) (G), and CD8^+^ T cells (CD69^+^CD3^+^CD8^+^) (H) in LNs. (I-L) MFI of CD69 in B cells (I), total T cells (J), CD4^+^ (K), CD8^+^ (L) T cells in the LNs. Data are shown as the mean ± SEM. n = 5 for the 0 μg group, n = 7 for the 5 μg and 10 μg groups. Statistics were calculated using unpaired one-way ANOVA with multiple comparison tests. *P < 0.05, **P < 0.01, ***P < 0.001; ns, not significant.


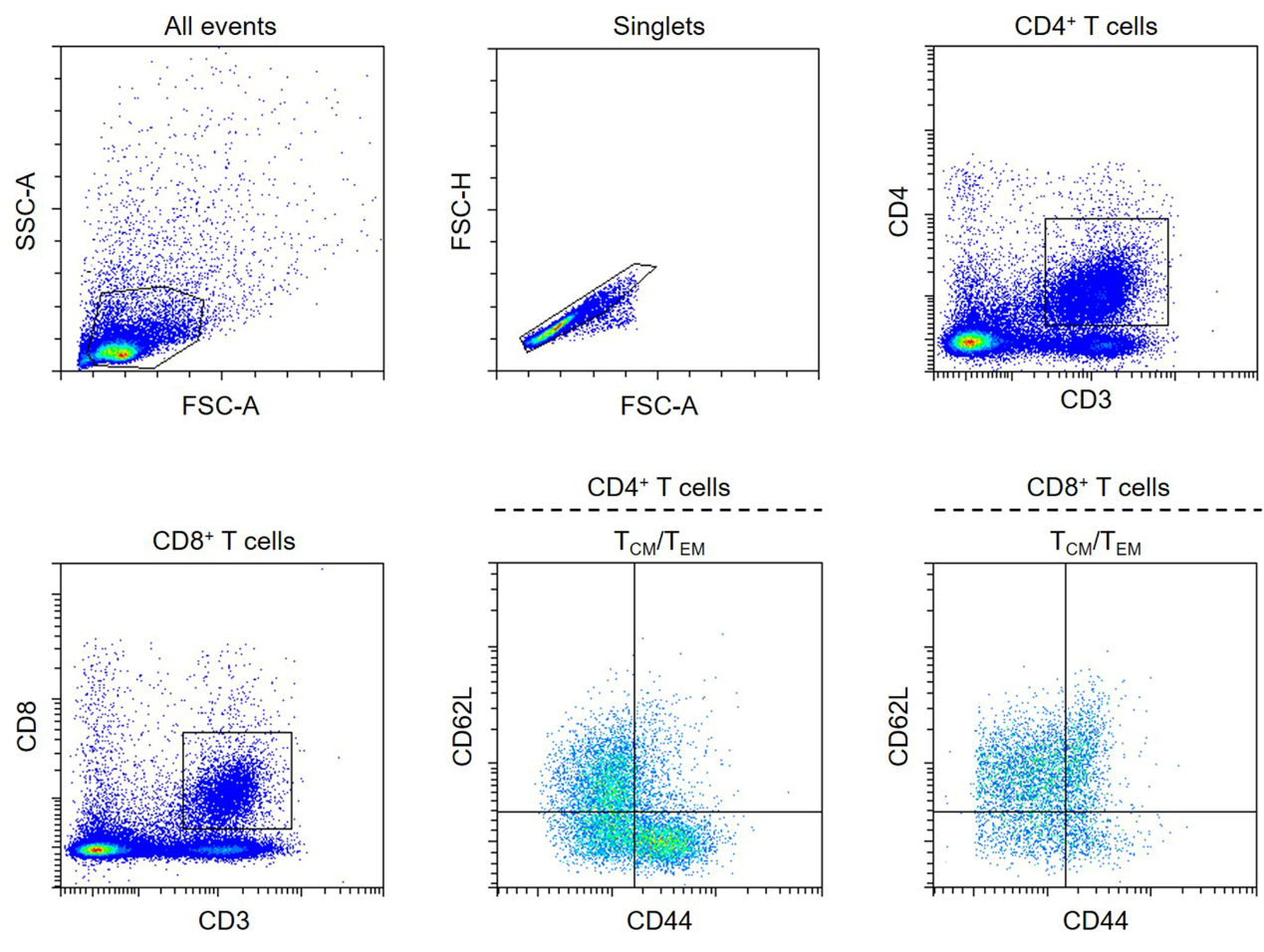


**FIGURE S10. The gating strategy for flow cytometry analysis of data is shown in Figure 2H-O.** Flow cytometry gating strategy for identification of effector memory T cells (T_EM_) and central memory T cells (T_CM_) in CD4^+^ T cells and CD8^+^ T cells.


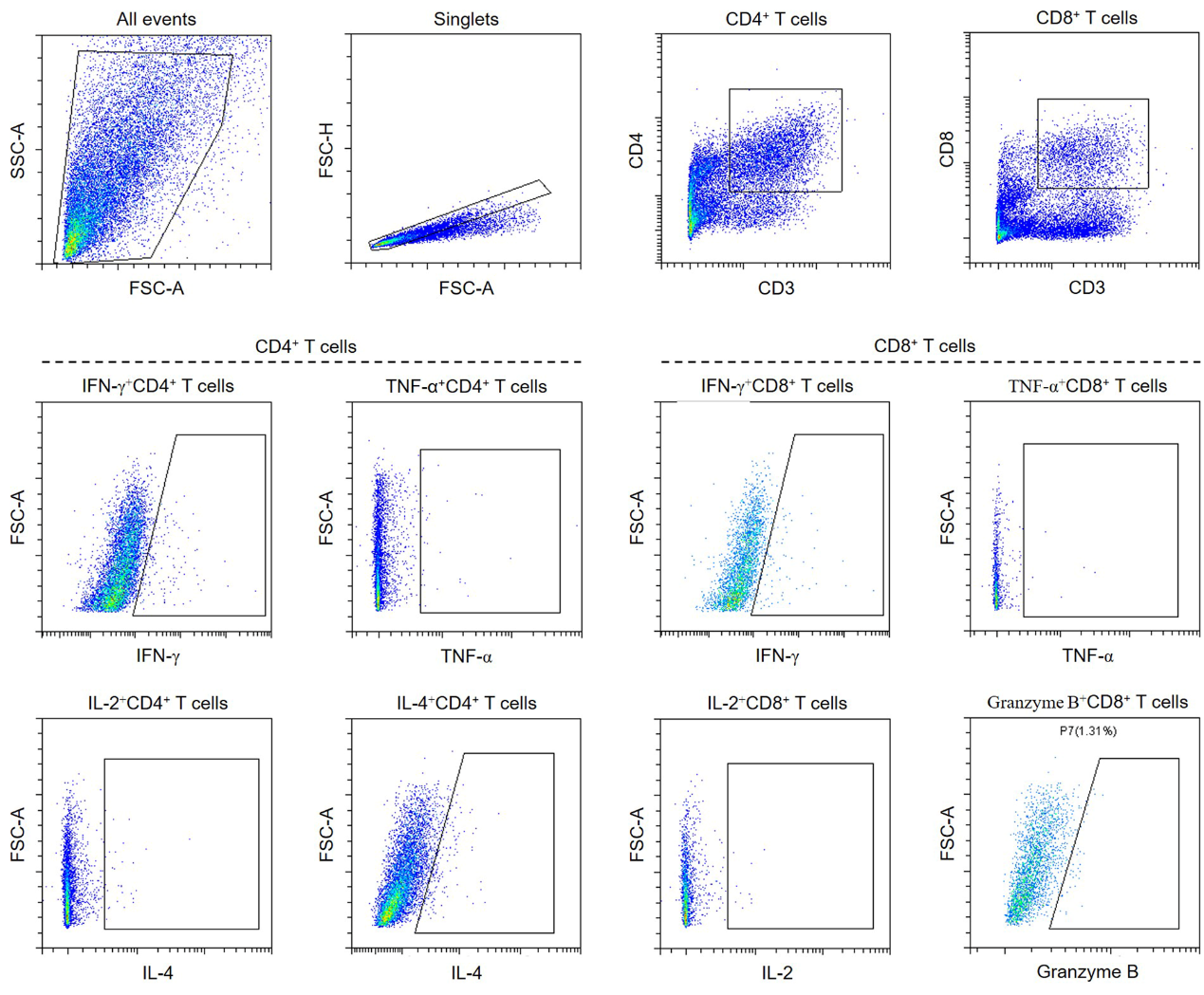


**FIGURE S11. The gating strategy for flow cytometry analysis of data is shown in Figure 3A-H.** Flow cytometry gating strategy for the identification of cytokine-positive cells in T cells.
